# Damage control surgery for grade IV blunt hepatic injury with multiple organ damage in a child: a case report

**DOI:** 10.1186/s40792-021-01348-8

**Published:** 2021-12-20

**Authors:** Jun Soma, Daisuke Ishii, Hisayuki Miyagi, Seiya Ishii, Keita Motoki, Hidemasa Kawabata, Shunta Ishitoya, Masahiro Hagiwara, Naohiro Kokita, Masatoshi Hirasawa

**Affiliations:** 1 Division of Pediatric Surgery, Department of Surgery, Asahikawa Medical University, 2-1-1, Midorigaokahigashi, Asahikawashi, Hokkaido 078-8510 Japan; 2 Division of Gastroenterology and Hematology/Oncology, Department of Medicine, Asahikawa Medical University, 2-1-1, Midorigaokahigashi, Asahikawashi, Hokkaido 078-8510 Japan; 3 Department of Radiology, Asahikawa Medical University, 2-1-1, Midorigaokahigashi, Asahikawashi, Hokkaido 078-8510 Japan; 4 Division of Hepato-Biliary-Pancreatic Surgery and Transplant Surgery, Department of Surgery, Asahikawa Medical University, 2-1-1, Midorigaokahigashi, Asahikawashi, Hokkaido 078-8510 Japan; 5 Department of Emergency Medicine, Asahikawa Medical University, 2-1-1, Midorigaokahigashi, Asahikawashi, Hokkaido 078-8510 Japan

**Keywords:** Blunt hepatic injury, Children, Damage control surgery, ABTHERA

## Abstract

**Background:**

Intra-abdominal hemorrhage caused by blunt hepatic injury is a major cause of morbidity and mortality in patients with abdominal trauma. Some of these patients require laparotomy, and rapid decision-making and life-saving surgery are essential. Damage control (DC) surgery is useful for treating children in critical situations. We performed this technique to treat an 8-year-old boy with grade IV blunt hepatic injury and multiple organ damage. This is the first report of the use of the ABTHERA Open Abdomen Negative Pressure Therapy System (KCI, now part of 3 M Company, San Antonio, TX, USA) for DC surgery to rescue a patient without neurological sequelae.

**Case presentation:**

An 8-year-old boy was brought to the emergency department of our hospital after being run over by a motor vehicle. He had grade IV blunt hepatic injury, thyroid injury, and bilateral hemopneumothorax. Although he was hemodynamically stable, the patient’s altered level of consciousness, the presence of a sign of peritoneal irritation, and suspicion of intestinal injury led us to perform exploratory laparotomy. As part of a DC strategy, we performed gauze packing to control hemorrhage from the liver and covered the abdomen with an ABTHERA Open Abdomen Negative Pressure Therapy System to improve the patient’s general condition. Eighteen days after admission, the patient was diagnosed with a biliary fistula, which improved with percutaneous and external drainage. He had no neurological sequelae and was discharged 102 days after injury.

**Conclusion:**

The DC strategy was effective in children with severe blunt hepatic injury. We opted to perform DC surgery because children have less hemodynamic reserve than adults, and we believe that using this strategy before the appearance of trauma triad of death could save lives and improve outcomes. During conservative management, it is important to adopt a multistage, flexible approach to achieve a good outcome.

## Background

Intra-abdominal hemorrhage caused by blunt hepatic injury is a major cause of morbidity and mortality in patients with abdominal trauma. In recent years, non-operative management has been considered the standard care for children with trauma because of improvements in resuscitation methods, diagnostic imaging, and pediatric intensive care facilities and the increased use of interventional radiology [1–3]. Despite this, some patients require laparotomy, and damage control (DC) surgery has been proven to be useful in such situations. Use of the DC strategy is considered in patients with hemorrhagic shock who show trauma triad of death of acidosis, coagulopathy, and hypothermia. The indications for DC surgery in children are the same as those in adults [4]. However, severe trauma in children is rare, and their anatomy and physiology are different from those of adults in many respects; therefore, there is no definitive evidence-based algorithm for the treatment of severe trauma in children.

We report the case of a child with severe blunt hepatic injury who underwent DC surgery and did not have any neurological sequelae. To our knowledge, this is the first reported case in which the ABTHERA Open Abdomen Negative Pressure Therapy System has been used in a child.

## Case presentation

The patient, an 8-year-old Japanese boy with the weight of 27 kg, was run over by a motor vehicle traveling at approximately 40 km/h when he was playing in the street. Emergency services were called, and a doctor car was dispatched. Examination performed when the doctor car reached the accident site revealed that the patient’s blood pressure (BP) was maintained at 112/73 mmHg, but his heart rate (HR) was increased (149 beats per minute). Focused assessment of sonography for trauma (FAST) revealed fluid collection in the bladder and ischio-rectal fossa, and fluid resuscitation was initiated after securing an intravenous line. The patient was transferred to the emergency department of our hospital approximately 40 min after the accident. On admission, he was able to speak, but was restless and complained of severe upper abdominal pain. His BP was 112/73 mmHg, HR was 140 beats per minute, respiratory rate was 44 breaths per minute, and percutaneous oxygen saturation was 100% on 10 L/min of oxygen via mask. His Glasgow Coma Scale score was 13.

**Fig. 1:**
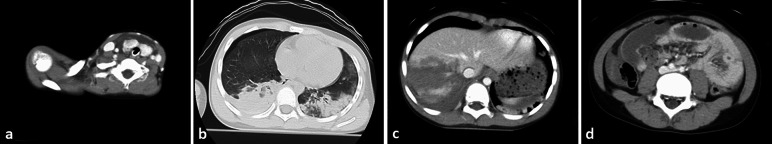
**a**–**d** Preoperative contrast-enhanced computed tomography images. Axial view shows irregularity and fluid collection around the thyroid gland, bilateral hemopneumothorax and pulmonary contusion, faint contrast effect with a band on the right lobe of the liver, and localized dilatation of the small intestine in multiple locations

**Fig. 2:**
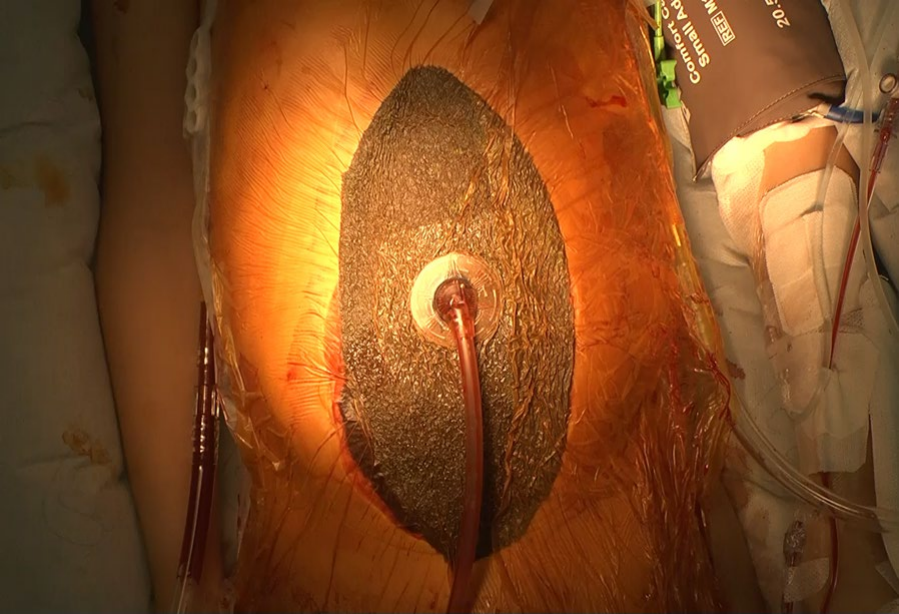
Image of the patient’s abdomen after damage control surgery. The abdomen has been temporarily covered with an ABTHERA Open Abdomen Negative Pressure Therapy System

FAST revealed fluid collection in the bladder, ischio-rectal fossa, and Morrison's fossa, which was suggestive of intra-abdominal hemorrhage. Contrast-enhanced computed tomography (CT) performed 17 min after admission with rapid intubation in the emergency room showed thyroid injury, bilateral hemopneumothorax and pulmonary contusion, and grade IV right hepatic lobe parenchymal injury [5] (Fig. 1a–c). Although there was no obvious free air, some intestinal dilatation was observed, suggestive of intestinal injury (Fig. 1d). No significant extravasation from the hepatic artery was observed. At this time, the patient’s serum hemoglobin level was 9.6 g/dL and his prothrombin time-international normalized ratio was 1.25 (Table 1). Red blood cells (RBCs) and fresh frozen plasma (FFP) were transfused. His hemodynamic condition stabilized with fluid resuscitation and blood transfusion. He had acidosis (pH 7.338) and a bicarbonate level of 19.9 mmol/L, base excess of − 4.9 mmol/L, and lactate level of 24 mmol/L. Although his hemodynamic condition was stable, the patient’s altered level of consciousness, the presence of signs of peritoneal irritation, and suspicion of intestinal injury led us to perform exploratory laparotomy. After thoracic cavity drains were placed bilaterally for treating the hemopneumothorax, the patient was transferred to the operating room 57 min after admission. On opening the abdomen, there was spillage of a large amount of hemorrhagic ascites fluid. A laceration was noted in the right lobe of the liver, and no intestinal injury was observed. As part of a DC strategy, we packed the right lobe of the liver from the ventral and dorsal sides using gauze. We covered the abdomen with an ABTHERA Open Abdomen Negative Pressure Therapy System (Fig. 2). The operative time was 23 min, total blood loss was 1185 mL, and total amount of blood transfused intraoperatively and preoperatively was 1120 mL of RBCs (eight units) and 240 mL of FFP (two units). The hemorrhage was gradually controlled with direct gauze packing. The anemia and coagulopathy improved; his serum hemoglobin level was 11.5 g/dL, prothrombin time was 14.3 s, and fibrinogen level was 304 mg/dL at 4 h after surgery.

**Table 1:** Laboratory data in the emergency room

*CBC*		*Chemistry*		
WBC	15,780 /μL	TP	5.3 g/dL	
RBC	357 × 10^4^ /μL	Alb	3.4 g/dL	
Hb	9.6 g/dL	T-Bil	0.4 mg/dL	
Hct	29%	AST	500 U/L	
PLT	27.4 /μL	ALT	300 U/L	
*Coagulation study*		AMY	46 U/L	
PT	14.3 s	ALP	232 U/L	
PT-INR	1.25	CK	255 U/L	
APTT	32.1 s	LDH	826 U/L	
AT-III	78%	BUN	15.4 mg/dL	
Fibrinogen	191 mg/dL	Cr	0.48 mg/dL	
D-dimer	17.9 μg/mL	Na	140 mEq/L	
*Arterial blood gas analysis*		K	3.0 mEq/L	
pH	7.338	Cl	108 mEq/L	
pCO2	38.1 mmHg	CRP	< 0.10 mg/dL	
pO2	114 mmHG			
HCO3	19.9 mmol/L			
BE	− 4.9 mmol/L			
Glu	270 mg/dL			
Lactate	24 mg/dL

Forty hours after DC surgery, we reperformed laparotomy, gently removed the gauze used for perihepatic packing, and directly closed the abdomen after confirming that there was no active bleeding from the liver and intestinal injury sites. The operative time was 20 min, and the total blood loss was 0 mL. The left and right thoracic drains were removed on the fifth and sixth days after injury, respectively. The patient was successfully extubated on day 7 of admission. His total length of intensive care unit (ICU) stay was 5 days.

**Fig. 3:**
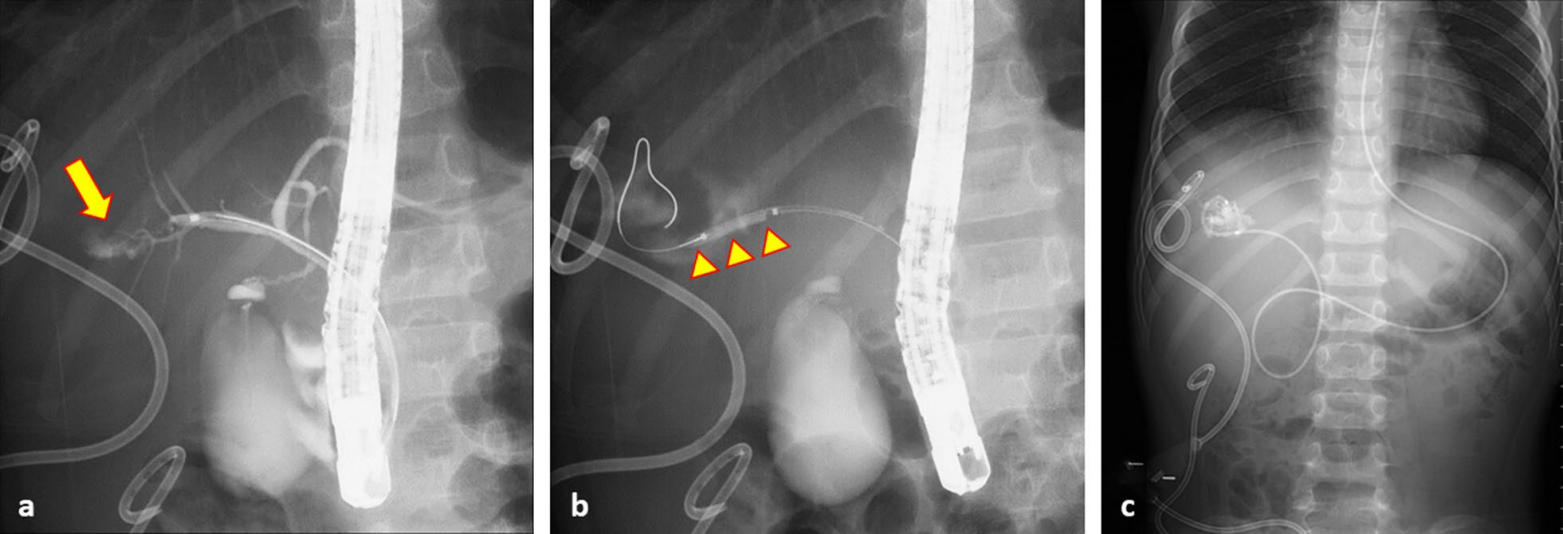
Abdominal X-ray. **a** Cholangiography using ERCP shows the bile leak from the bile duct of B6 (yellow arrow). **b** Bile duct dilatation using the balloon of 3 mm in diameter (yellow arrowheads). **c** Endoscopic nasobiliary drainage (ENBD) and percutaneous drainage of the traumatic biliary fistula at the right subdiaphragmatic and subhepatic regions

We continued to evaluate the hepatic injury using abdominal ultrasonography and CT. The patient experienced abdominal distension caused by ascites fluid and right lateral abdomen pain 18 days after injury. An abdominal drain was inserted at the site of lateral abdomen pain. Abdominal ultrasonography revealed fluid collection in the right infradiaphragmatic region. He had high total and direct bilirubin levels and was diagnosed with a biliary fistula, with more than 400 mL of fluid drainage per day. Magnetic resonance cholangiopancreatography revealed injury to the posterior branch of the bile duct 25 days after the injury. The biliary fistula showed poor improvement with percutaneous drainage. Endoscopic nasobiliary drainage (ENBD) was performed using endoscopic retrograde cholangiopancreatography (ERCP) after bile duct dilatation 59 days after the injury (Fig. 3). The volume of bile drained through the percutaneous drainage tube was approximately 400 mL/day for the first 2 weeks, and about 100 mL/day thereafter. After ENBD implantation, the volume gradually decreased. At the time of discharge from the hospital, almost no drainage was observed. The patient had no neurological sequelae and was discharged from the hospital 102 days after the injury. The drain was removed during an outpatient visit after cessation of the bile leak.

## Discussion

The liver is the most commonly injured intra-abdominal solid organ [6]. Hepatic injury occurs in 15–20% of all children with abdominal trauma [7]. The World Society of Emergency Surgery has classified liver injuries into four groups based on the Organ Injury Scaling Committee of the American Association for the Surgery of Trauma classification and hemodynamic condition [5]. In addition to injury severity, the hemodynamic status of the patient is important for determining the treatment plan for traumatic liver injury. Exploratory laparotomy is required in patients with multiple organ damage, such as intestinal injury [8, 9].

Contrast-enhanced CT is useful for detecting abdominal compartment syndrome and intra-abdominal and retroperitoneal injuries. However, injury severity assessed using contrast-enhanced CT does not necessarily correlate with the need for emergency surgery [10]. It is important to comprehensively evaluate the patient based on clinical and imaging findings, resuscitate the patient immediately and aggressively, and perform exploratory laparotomy if needed. In our patient, although the hemodynamic condition was stable, an altered level of consciousness, a sign of peritoneal irritation, and suspicion of intestinal injury led us to perform exploratory laparotomy.

Rapid transfusion of a large amount of blood to maintain vital sign stability in a patient with massive hemorrhage can results in coagulopathy. The occurrence of coagulopathy is statistically correlated with hypothermia and acidosis [11]. Trauma triad of death except hypothermia were seen in our patient. In general, DC surgery is considered when a patient shows all components of trauma triad of death. The mortality rate of adults with traumatic hepatic injury who have a bleeding tendency has been reported to be more than 85% [12]. We opted for a DC strategy because children have less hemodynamic reserve than adults, and we believe that using this strategy before the appearance of trauma triad of death could save lives and improve outcomes. DC surgery involves the initial control of bleeding and contamination, followed by gauze packing and rapid wound closure [13]. The risk of infection increases if the packed gauze is retained for more than 72 h [14]. Therefore, after the patient's general condition stabilized following within 72 h of ICU admission, we reperformed laparotomy to re-evaluate the injured region and repair it if needed. DC surgery with gauze packing has been reported to be useful in children with severe intra-abdominal hemorrhage [15]. Perihepatic packing controls profuse hemorrhage in up to 80% of patients undergoing laparotomy and allows for intraoperative resuscitation [13, 16–19]. Three effective options for the management of hepatic vein injuries: tamponade with liver packing, direct repair, and hepatic resection [5]. Liver packing is the safest method of temporarily dealing with severe venous injuries [20–22]. Temporary abdominal closure may be indicated if the risk of abdominal compartment syndrome is high or in situations in which a “second-look” operation is needed [23, 24]. The ABTHERA Open Abdomen Negative Pressure Therapy System can be easily adapted to the size of the open abdominal wound and is therefore considered safe to use in children, who have a small build. General considerations for pediatric open abdominal management include increased risk of infection due to prolonged exposure and difficulty with volume control due to small blood volume and rapid heat loss [4, 15].

The incidence of biliary complications after blunt hepatic trauma has been reported to be 2.8–7.4% [25]. In addition to high-grade injury, centrally located liver injuries and early transcatheter arterial embolization are significant risk factors for major bile duct injury [26, 27]. There are few reports on the incidence of biliary fistulas in children; therefore, their management remains controversial. In general, external fistula and percutaneous drainage is effective and safe for the treatment of bile leaks [28]. Our patient’s biliary fistula was insufficiently controlled with percutaneous drainage. We performed ERCP and ENBD implantation after bile duct dilatation using the balloon. This reduced the internal pressure in the bile duct and promoted spontaneous recovery of the injured region [29]. In addition, the peripheral bile duct was not observed when we performed ERCP for the bile leak. Since there was no dilatation of the peripheral bile duct, we considered that there was no peripheral intrahepatic drainage failure. Therefore, bridging of the laceration site was not required in this case. Conservative management is useful for traumatic biliary fistulas, and it is important that a multistage, flexible approach is adopted to achieve favorable success.

## Conclusions

The DC strategy can be effective even in children with severe blunt hepatic injury. Although non-operative management should always be considered as the first-choice treatment in children, the best strategy, which may include exploratory laparotomy, should be determined after considering the clinical symptoms, grade of injury, and other organ injuries. During conservative management, it is important to adopt a multistage, flexible approach to achieve good outcomes.

## Data Availability

Not applicable.

## Abbreviations

DC: Damage control
BP: Blood pressure
HR: Heart rate
FAST: Focused assessment of sonography for trauma
CT: Computed tomography
RBCs: Red blood cells
FFP: Fresh frozen plasma
ICU: Intensive care unit
ENBD: Endoscopic nasobiliary drainage
ERCP: Endoscopic retrograde cholangiopancreatography
